# Draft Genome Sequence of *Caenibacillus caldisaponilyticus* B157^T^, a Thermophilic and Phospholipase-Producing Bacterium Isolated from Acidulocompost

**DOI:** 10.1128/genomeA.00089-17

**Published:** 2017-03-30

**Authors:** Yoshiyuki Tsujimoto, Ryo Saito, Takehiko Sahara, Nobutada Kimura, Naoki Tsuruoka, Yasushi Shigeri, Kunihiko Watanabe

**Affiliations:** a Graduate School of Life and Environmental Sciences, Kyoto Prefectural University, Shimogamo, Sakyo, Kyoto, Japan; b Bioproduction Research Institute, National Institute of Advanced Industrial Science and Technology, Higashi, Tsukuba, Ibaraki, Japan; c Health Research Institute, National Institute of Advanced Industrial Science and Technology, Midorigaoka, Ikeda, Japan

## Abstract

*Caenibacillus caldisaponilyticus* B157^T^ (= NBRC 111400^T^ = DSM 101100^T^), in the family *Sporolactobacillaceae*, was isolated from acidulocompost as a thermophilic and phospholipid-degrading bacterium. Here, we report the 3.36-Mb draft genome sequence, with a G+C content of 51.8%, to provide the genetic information coding for phospholipases.

## GENOME ANNOUNCEMENT

Acidulocomposting is an effective method for degrading otherwise-unusable organic materials under acidic conditions (pH 3.5 to 6.5) at approximately 50 to 70°C, and various bacteria grow throughout the composting process (1–3). However, little information was previously reported on novel bacteria from acidulocompost. *Caenibacillus caldisaponilyticus* B157^T^ (= NBRC 111400^T^ = DSM 101100^T^) was isolated as a thermophilic and phospholipid-degrading bacterium from acidulocompost at Oarai Aquarium in Ibaraki, Japan, and thereafter recognized as a novel species of a new genus in the family *Sporolactobacillaceae* (4). The organism is Gram positive, aerobic, spore forming, and rod shaped. Growth was observed to occur at 40 to 65°C and pH 4.8 to 8.1.

Strain B157^T^ produced a thermostable extracellular phospholipase A (PLA), which degraded phospholipids to generate lysophospholipids and free fatty acids (data not shown). Although thermostable PLAs are expected to be of use in the oil and food industries, a limited number of such enzymes have been purified and characterized so far (5, 6). Therefore, the enzyme from strain B157^T^ is of interest from the viewpoints of both basic and applied sciences. However, the PLA has failed to be purified and identified due to nonspecific binding of the enzyme to the chromatographic resins. Accordingly, in order to identify the corresponding gene, we determined the draft genome sequence using the Ion PGM system (Thermo Fisher Scientific, Waltham, MA, USA). The resulting 917,608 reads were assembled *de novo* using the MIRA 4.0.5 software program (7) into 181 contigs. The *N*_50_ contig length was 60,783 bp. The draft genome was annotated by the Rapid Annotations using Subsystems Technology (RAST) version 2.0 online annotation server (8–10).

The draft genome included 3,356,119 bases with a G+C content of 51.8%. The B157^T^ genome included 3,924 protein-coding genes, 65 tRNA genes, and 23 rRNA genes. Although strain B157^T^ produced an extracellular PLA, there was no candidate screened for PLA activity by RAST analysis. Moreover, the Microbial Genome Annotation Pipeline (MiGAP) version 2.19 (11) failed to identify any PLA genes. Therefore, we broadened the search for four homologous enzymes: patatin-like phospholipases (Patatins), lysophospholipase-like phospholipases (LysoPLs), phospholipase/carboxylesterases (CEs), excluding phospholipases C and D, and lipases. This is because some of these enzymes are known to exhibit PLA activity (5, 6, 12). Then, we carried out the screening of those four enzymes in the genome using two software programs, RAST and MiGAP. As a result, a total of seven genes were newly identified: three genes were annotated as Patatin, LysoPL, and CE, respectively, two genes as LysoPL or lipase, one gene as LysoPL or CE, and one gene as LysoPL or protein with unknown function. We are investigating whether the gene products of those seven candidates have PLA activities by expressing them in *Escherichia coli*. This approach reveals the true PLA gene in the genome, and the subsequent study provides novel and structural information of bacterial PLA from the viewpoints of PLA evolution and classification.

### Accession number(s).

The draft genome sequence of *C. caldisaponilyticus* B157^T^ was deposited in the DDBJ/EMBL/GenBank database under accession numbers BDDQ01000001 to BDDQ01000181.
